# A high-resolution 7 Tesla resting-state fMRI dataset optimized for studying the subcortex

**DOI:** 10.1016/j.dib.2024.110668

**Published:** 2024-06-24

**Authors:** Josephine M. Groot, Steven Miletic, Scott J.S. Isherwood, Desmond H.Y. Tse, Sarah Habli, Asta K. Håberg, Pierre-Louis Bazin, Matthias Mittner, Birte U. Forstmann

**Affiliations:** a Integrative Model-based Cognitive Neuroscience research unit, University of Amsterdam, Amsterdam, 1001 NK, the Netherlands; b Department of Psychology, UiT – The Arctic University of Norway, Tromsø, 9037, Norway; c Department of Neuropsychology and Psychopharmacology, Maastricht University, Maastricht, MD 6200, the Netherlands; d Department of Psychology, Norwegian University of Science and Technology, Trondheim, 8900, Norway; e Department of Neuromedicine and Movement Science, Norwegian University of Science and Technology, Trondheim, 8900, Norway; f Department of Radiology and Nuclear Medicine, St. Olavs Hospital, Trondheim, 7006, Norway; g Institute of Psychology, Leiden University, Leiden, 2300, the Netherlands; h Full brain picture Analytics, 2332 XB Leiden, the Netherlands

**Keywords:** Ultra-high field, BOLD, MP2RAGE, fMRIPrep, Cardiac, Respiration

## Abstract

To achieve a comprehensive understanding of spontaneous brain dynamics in humans, *in vivo* acquisition of intrinsic activity across both cortical and subcortical regions is necessary. Here we present advanced whole-brain, resting-state functional magnetic resonance imaging (rs-fMRI) data acquired at 7 Tesla with 1.5 mm isotropic voxel resolution. Functional images were obtained from 56 healthy adults (33 females, ages 19–39 years) in two runs of 15 min eyes-open wakeful rest. The high spatial resolution and short echo times of the multiband echo-planar imaging (EPI) protocol optimizes blood oxygen level-dependent (BOLD)-sensitivity for the subcortex while concurrent respiratory and cardiac measures enable retrospective correction of physiological noise, resulting in data that is highly suitable for researchers interested in subcortical BOLD signal. Functional timeseries were coregistered to high-resolution T1-weighted structural data (0.75 mm isotropic voxels) acquired during the same scanning session. To accommodate data reutilization, functional and structural images were formatted to the Brain Imaging Data Structure (BIDS) and preprocessed with fMRIPrep.

Specifications Table

**Table:** 

Subject	Neuroscience: Cognitive
Specific subject area	Ultra-high field human resting-state functional magnetic resonance imaging.
Type of data	Structural brain images (3D NIfTI [.nii.gz]) Functional brain images (4D NIfTI [.nii.gz]) Metadata (Javascript object notation [.json]) Confounds timeseries (tab-separated values [.tsv]) Image transforms (HDF5 [.h5], textfile [.txt]) Cardiac and respiratory recordings (Siemens logfiles [.log]) Participant demographics (comma-separated values [.csv]) Temporal signal to noise ratio values (Figure)
Data collection	As part of a larger data collection initiative, 56 healthy adult volunteers (33 females, ages 19–39 years) were scanned with a Siemens 7 Tesla MAGNETOM Terra system with a 32-channel phased-array head coil. Whole-brain functional images were acquired in two multiband EPI-BOLD runs of 15 min each during eyes-open wakeful rest (TR=1380 ms, TE=14 ms, flip angle=60°, GRAPPA factor=3, multiband acceleration factor=2, voxel size=1.5 mm isotropic). An additional scan was performed with opposite phase encoding direction to measure and correct for susceptibility-induced field distortions. Concurrent cardiac and respiratory data were recorded to facilitate retrospective correction of physiological noise. Structural T1-weighted images were obtained from a high-resolution MP2RAGE sequence (TR=4300 ms; TI_1,2_ = 840, 2370 ms; flip angle_1,2_ = 5, 6°; TE=1.99 ms, voxel size=0.75 mm isotropic) in the same scanning session.
Data source location	Norwegian 7T MR Center, St. Olavs Hospital, Trondheim, 7006, Norway (https://www.ntnu.edu/mh/7tmr). Latitude and longitude: 63°25′15.8″N, 10°23′16.8″E
Data accessibility	Repository name: Figshare Data identification number: 10.21942/uva.25690692 Direct URL to data: https://doi.org/10.21942/uva.25690692.v1
Related research article	J.M. Groot, S. Miletic, S.J.S. Isherwood, D.H.Y. Tse, S. Habli, A.K. Håberg, B.U. Forstmann, P-L. Bazin, M. Mittner, Echoes from Intrinsic Connectivity Networks in the Subcortex, J. Neurosci. 43 (2023) 6609–6618.

## 1 Value of the Data

• The fMRI protocol is tailored toward subcortical imaging at ultra-high field strength (7 Tesla) and is of interest to researchers studying intrinsic dynamics in cortical and subcortical regions.
• BOLD timeseries were collected in two runs (total 30 min) of eyes-open wakeful rest and are coregistered to high-resolution T1-weighted structural data.
• Concurrently acquired cardiac and respiratory measures are shared in raw format and allow application of post-scanning denoising procedures (e.g., RETROICOR) to correct for physiological sources of noise that are prominent in the subcortex.
• Imaging data and corresponding metadata are anonymized, converted to BIDS format, and preprocessed with fMRIPrep to facilitate further usage.

## 2 Background

The acquisition of whole-brain resting-state fMRI data are necessary for achieving a comprehensive understanding of the fundamental organization of brain activity in humans. Although important cognitive and attentional processes rely on brain networks that encompass both cortical and subcortical regions, our understanding of the intrinsic architecture of numerous nuclei in the subcortex remains limited. While many resting-state fMRI datasets are currently available, the presented data were tailored to imaging cortical as well as subcortical functional dynamics. Optimizing the BOLD-sensitivity of the fMRI protocol involved increasing spatial resolution and aligning the echo time (TE) to the shortened T2* relaxation times of many iron-rich subcortical nuclei. The data can therefore be leveraged to improve our understanding of the complex connectivity patterns among subcortical structures as well as between the subcortex and the rest of the brain, providing vital information for charting the principles of whole-brain organization.

## 3 Data Description

As part of a larger, multi-session data collection initiative, healthy adult volunteers were scanned in four scanning sessions on separate days. Here, we present data from the first scanning session, including whole-brain structural, functional, and physiological data obtained during eyes-open wakeful rest. The dataset can be downloaded from Figshare [1] and has been previously used in a study investigating subcortical functional architecture and connectivity to cortical resting-state networks [2].

**Fig. 1:**
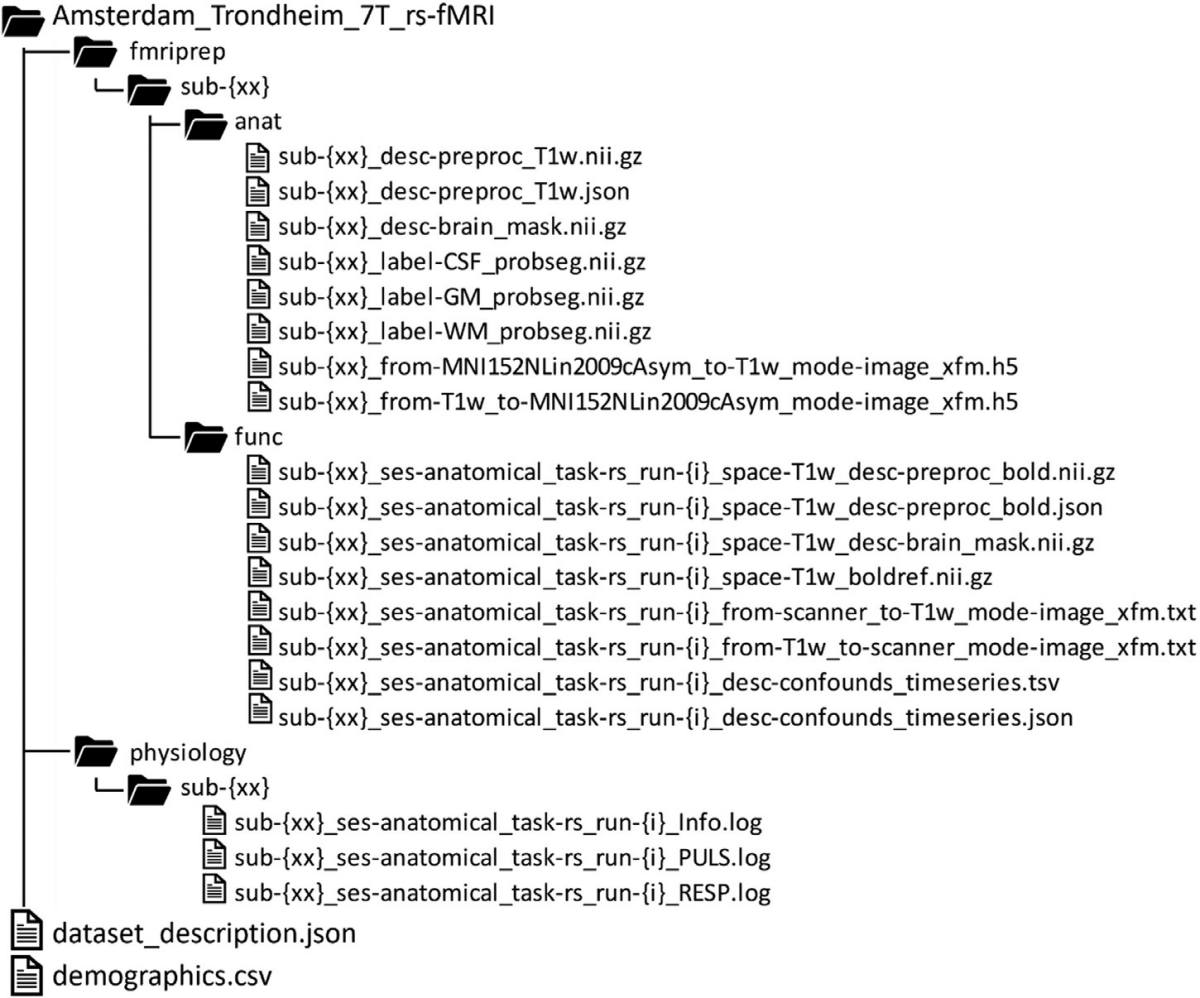
Overview of the data structure.

The data structure is presented in Fig. 1. Every file has a double-digit participant identifier and the functional and physiological data have an additional run identifier (i.e., 1 or 2). The root directory contains a ‘dataset_description.json’ file with general information and a ‘demographics.csv’ file with participant's sex and age-groups. Individual anatomical fMRIPrep derivatives in the [fmriprep/sub-{xx}/anat/] directories include: the preprocessed T1-weighted (T1w) images and corresponding metadata, brain masks, automatic segmentations of CSF, white matter, and grey matter, and transforms to and from the ICBM 152 Nonlinear Assymetrical template version 2009c (MNI152Nlin2009cAsym). Functional fMRIPrep derivatives for each of the two runs in the [fmriprep/sub-{xx}/func/] directories include: the preprocessed BOLD images and corresponding metadata, brain masks, BOLD reference volumes, coregistrations to and from the T1w image, and confounds timeseries. Raw physiological recordings for each of the two BOLD runs are in the [physiology/sub-{xx}/] directories to accommodate optional future processing with MATLAB PhysIO toolbox [3], which at the time of writing does not work with BIDS-formatted data. The [*_Info.log] files contain general acquisition information and the timing of measurement per volume and slice in terms of ‘ACQ_START_TICS’ and ‘ACQ_FINISH_TICS’, where one tic equals 2.5 ms and the acquisition is mentioned in the number of tics since midnight. The [*_PULS.log] contains heart rates collected at 200 Hz frequency (i.e., one sample every two tics) and the [*_RESP.log] contains respiratory data collected at 50 Hz frequency (i.e., one sample every eight tics)*.*

## 4 Experimental Design, Materials and Methods

### 4.1 Participants

Participants were 56 healthy adults (33 females) between the ages of 19 and 39 (*M* = 26.16, *SD*=5.46) that were recruited from the Norwegian University of Science and Technology (NTNU). Inclusion criteria included: no self-reported (history of) psychiatric or neurological disease, normal or corrected-to-normal vision, and no contra-indications for MRI such as metal implants or pregnancy. Written informed consent was obtained from all participants prior to any experimental procedure. Participants were scanned in four sessions on separate days, including one session with structural scans and resting-state fMRI (presented here) followed by three experimental fMRI sessions with various decision-making paradigms. Participation was compensated with gift card certificates.

### 4.2 Data acquisition

Participants were scanned with a MAGNETOM Terra 7 Tesla MRI system (Siemens Healthineers, Germany) with a scanner gradient strength of 80 mT/m at 200 T/m/s, using a 32-channel phased array head coil (Nova Medical Inc, USA). During scanning, participants were instructed to stay awake and keep their gaze fixated on a static central fixation cross that was presented on a gray background. We used a Magnetization Prepared 2 Rapid Acquisition Gradient Echoes (MP2RAGE) [4]. to obtain whole-brain structural images in 224 sagittal slices with 0.75 mm isotropic voxel resolution: TR=4300 ms; TI_1,2_ = 840, 2370 ms; flip angle_1,2_ = 5, 6°; TE=1.99 ms, FOV=240×240×168 mm, bandwith=250 Hz/Px. Acquisition time (TA) was 9:25 min. Functional (BOLD) timeseries were acquired using a single-echo multiband 2D gradient-echo EPI sequence (release R016b) developed by the Center for Magnetic Resonance Research (CMRR, University of Minnesota, cmrr.umn.edu/multiband). The echo time (TE) was tailored to optimize BOLD-contrast sensitivity to the relatively short T2* relaxation times of iron-rich nuclei the human subcortex compared to the cortex [5,6]. While it has been argued that multi-echo protocols can avoid tradeoffs in BOLD sensitivity between the cortex and subcortex, we recently demonstrated that despite the low TE, the single-echo protocol produced higher temporal signal-to-noise (SNR) across both subcortical and cortical regions compared to the multi-echo protocol, while also retaining adequate contrast-to-noise ratio (CNR) in the cortex [6]. Functional images were acquired in 82 transverse slice per volume with 1.5 mm isotropic voxel resolution: TR=1380 ms, TE=14 ms, flip angle=60°, in-plane acceleration (GRAPPA) factor=3, multiband acceleration factor=2, partial Fourier=6/8, FOV=192×192×132 mm, multi-slice mode=interleaved, matrix size=128×128, bandwith=1446 Hz/Px, echo spacing=0.8 ms, phase encoding (PE) direction=Anterior > Posterior, TA per run=15:37. Each of the two BOLD runs was directly followed by a short GE-EPI sequence (TA=0:43) with opposite PE direction (Posterior>Anterior) to measure and correct for susceptibility-induced field distortions. Heart rate data were collected with a photoplethysmograph at a 200 Hz sampling frequency, while respiration was recorded at a 50 Hz sampling rate using a waistbelt. Due to technical issues, one subject (sub-45) has missing cardiac data for the second BOLD run (*_run-2_PULS.log).

### 4.3 Conversion and preprocessing

**Fig. 2:**
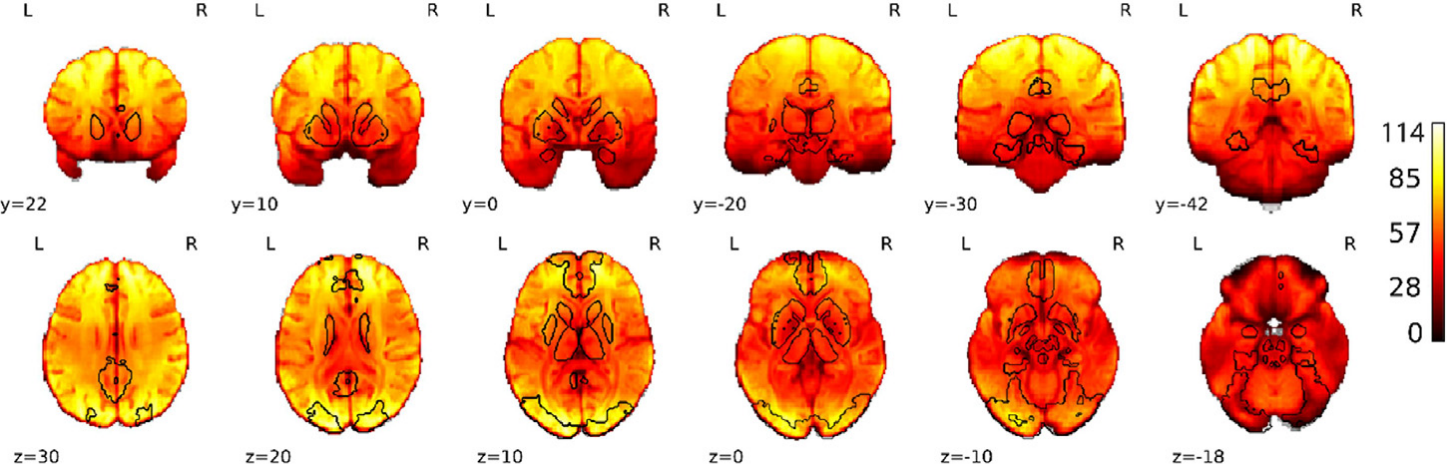
Whole-brain, voxel-wise temporal SNR values. For a subset of subjects (*n* = 40), temporal SNR values were calculated for both runs as the ratio of the mean and standard deviation of the resting-state timeseries after temporal high-pass filtering (1/128 s). Individual temporal SNR maps were then registered to standard space (MNI152Nlin2009cAsym) and averaged to create a group-level map. Black contours indicate the outlines of binary masks for cortical and subcortical regions of interest (for details we refer to our related research article; [2]). Mean ± standard deviations of temporal SNR values for individual subcortical regions of interest: thalamus (47.94±6.31), striatum (52.17±8.11), globus pallidus externa (35.44±5.58), globus pallidus interna (34.18±4.39), subthalamic nucleus (32.30±4.25), claustrum (59.12±4.71), hippocampus (37.84±10.44), amygdala (39.89±7.22), substantia nigra (31.51±5.32), red nucleus (33.75±3.05), ventral tegmental area (37.68±3.05), periaqueductal gray (32.37±10.56), locus coeruleus (39.01±7.31), and pedunculopontine nucleus (40.00±3.29). Copied from Extended Data Fig. 2–1 [2].

Raw DICOM files were converted to NIfTI using dcm2niiX v1.0.20181125 [7]. NIfTIs were formatted into BIDS v1.4.1 with a custom Python script. The structural images were defaced using PyDeface v2.0.2. To facilitate fMRIPrep-based preprocessing, background noise suppression was performed according to the robust T1w image method [8] by recomputing the T1w images based on the second inversion (inv2) of the MP2RAGE with nilearn.image.math_img using the following formula: T1w × (inv2/(inv2+100)). The offset value (100) was chosen based on visual inspection of the results. Images were then preprocessed with fMRIPrep v20.2.6 [9] based on Nipype v1.7.0 [10]. First, structural images were corrected for intensity non-uniformity with N4BiasFieldCorrection [11] in ANTs (v2.3.3) [12] and skull-stripped with a Nipype implementation of the antsBrainExtraction workflow using the OASIS30ANTs target template. Then, tissue segmentation for CSF, WM, and GM was performed with FAST (FSL v5.0.9) [13] and brain surfaces were reconstructed with recon-all (FreeSurfer v6.0.1) [14]. Finally, structural images were normalized to standard MNI152Nlin2009cAsym space through non-linear registration with antsRegistration. For each functional run, a fieldmap was first generated based on the EPI references with opposing phase-encoding directions using 3dQwarp in AFNI [15] and used to calculate an EPI reference image corrected for susceptibility distortions. This reference image was coregistered to the T1w reference using FreeSurfer's boundary-based registration (bbregister) [16] with six degrees of freedom. Head-motion parameters (rotation and translation) were estimated prior to spatiotemporal filtering with mcflirt from FSL [17] and slice-time correction to half the acquisition range (0.674 s) with 3dTshift from AFNI (Cox & Hyde 1997). The BOLD timeseries were then resampled to native space by applying a single, composite transform to correct for head-motion and susceptibility distortions. Based on this preprocessed timeseries, several confounding timeseries were calculated: region-wise averaged signals (global_signal, csf, white_matter) and their temporal derivatives and quadratic terms to enable further preprocessing (columns with ‘_derivative1’ and ‘_power2’ suffixes), bulk head motion (‘framewise_displacement’), relative bulk head motion using the RMS approach (‘rmsd’), the derivative of RMS variance over voxels (‘dvars’) and its standardized version (‘std_dvars’), motion spikes (columns with ‘motion_outlier_’ prefixes), six head translation and rotation parameters (columns with ‘trans_’ and ‘rot_’ prefixes), discrete cosine transform regressors (columns with ‘cosine_’ prefixes), and anatomical and temporal component-based noise correction regressors (columns with ‘t_comp_cor_’ and ‘a_comp_cor_’ prefixes). To provide an indication of data quality, we present averaged whole-brain, voxel-wise temporal SNR values and mean temporal SNR for fourteen subcortical regions in Fig. 2 (copied from our related research article; [2]).

## Limitations

• Given the relatively short TE of the single-echo protocol (TE=14 ms), BOLD sensitivity is tailored to iron-rich nuclei in the subcortex and may result in less optimal CNR for cortical regions compared to BOLD protocols with longer TE.
• In our data, we observe relatively reduced temporal SNR in the temporal cortex compared to other cortical regions. This artifact is likely attributed to magnetic field inhomogeneities due to local differences in magnetic susceptibility in these regions, potentially leading to biases in estimating the intrinsic dynamics of the temporal lobe.
• The temporal resolution of our data (TR=1380 ms) – while sufficient for capturing fluctuations in resting-state networks – may not be suitable for researchers interested in resolving neural dynamics that at higher frequencies.

## Ethics Statement

Data collection with human participants was ethically approved by the Ethics Review Board of the Faculty of Social and Behavioral Sciences of the University of Amsterdam (reference: 2021-BC-13146) and the Regional Committees for Medical and Health Related Research Ethics of Central Norway (reference: 116630). Participants provided written informed consent prior to data collection and all shared data were anonymized to ensure the privacy of those who participated.

## CRediT authorship contribution statement

**Josephine M. Groot:** Data curation, Formal analysis, Writing – original draft. **Steven Miletic:** Methodology, Formal analysis, Validation, Writing – review & editing. **Scott J.S. Isherwood:** Investigation, Formal analysis. **Desmond H.Y. Tse:** Methodology, Data curation. **Sarah Habli:** Investigation, Data curation. **Asta K. Håberg:** Methodology, Resources. **Pierre-Louis Bazin:** Conceptualization, Software, Writing – review & editing. **Matthias Mittner:** Conceptualization, Supervision, Writing – review & editing. **Birte U. Forstmann:** Conceptualization, Funding acquisition, Project administration, Writing – review & editing.

## Data Availability

Amsterdam_Trondheim_7T_rs-fMRI (Original data) (Figshare).
